# Recent advances in understanding and treating scabies

**DOI:** 10.12703/r/10-28

**Published:** 2021-03-11

**Authors:** Emily Welch, Lucia Romani, Margot J Whitfeld

**Affiliations:** 1 St Vincent’s Hospital, University of New South Wales, Sydney, Australia; 2 Kirby Institute, Sydney, Australia

**Keywords:** Scabies, impetigo, crusted scabies, tropical disease, mite biology, developing countries, disease control, drug therapy, mass drug administration, ivermectin, moxidectin, permethrin

## Abstract

Scabies is an infestation of the skin caused by the mite *Sarcoptes scabiei.* In 2017, scabies was recognised by the World Health Organisation as a disease of public importance and was consequently added to the list of neglected tropical diseases. An estimated 200 million people currently have scabies worldwide. Scabies is endemic in many developing countries, with the highest prevalence being in hot, humid climates such as the Pacific and Latin American regions. Scabies causes a host immune response which is intensely itchy. Scratching of the lesions can lead to secondary bacterial infections of the skin, such as impetigo, most commonly caused by *Streptococcus pyogenes* or *Staphylococcus aureus*. This can have fatal consequences, such as septicaemia, glomerulonephritis, and rheumatic heart disease. Advances over the past 5 years indicate that mass drug administration is an effective strategy to treat scabies. This review will outline advances in the mite biology, epidemiological understanding, diagnosis, and treatment of scabies.

## Introduction

Since 2017, scabies has been recognised by the World Health Organization (WHO) as one of the most important neglected tropical diseases (NTDs)^1^. Scabies not only has significant health consequences but also has profound psychosocial effects such as itching, insomnia, loss of productivity, poor quality of life, and in one study resulted in a feeling of shame in 77% of sufferers^2,3^. In March 2018, the WHO NTD Global Working Group on Monitoring and Evaluation recommended that the global burden of scabies be established, diagnostic criteria be produced, and interim guidelines for public health interventions be introduced^4^. Throughout this review, recent advances in the epidemiology, diagnosis, and management of scabies will be described.

## Scabies epidemiology

Scabies is an intensely itchy parasitic disease of the skin caused by microscopic scabies mites that are transmitted from person to person via close contact. Scabies is endemic to resource-scarce communities where overcrowding and poverty are more common. As a result of poor access to treatment combined with domestic crowding, transmission of the mite can become widespread. Those who live in these endemic areas and seek treatment commonly experience rapid re-infestation when they come into contact with untreated households^5^. The economic burden for these communities is considerable, with direct costs relating to medications, sick days, and hospitalisation^6^.

Scabies occurs worldwide, with the highest prevalence documented in countries with hot, tropical climates^7,8^. The Pacific islands, Central America, and the indigenous population of Northern Australia have the highest documented levels. In Fiji, a survey reported that over one-third of residents had scabies and more than one in two children were affected^6^. Although more research is needed, the reported prevalence of scabies in Europe and Middle Eastern countries is low (<2.2%)^8^. It is not known whether this is purely due to socioeconomic factors or whether the scabies mite thrives in humid climates. In developed countries, scabies occurs sporadically, or at higher levels in situations of close living, such as institutions including schools, aged care facilities, prisons, and refugee camps, as well as in the homeless^6,9^. It can also be spread primarily through sexual transmission, again due to close contact^6^. Scabies was the cause of 58% of infectious disease/dermatology consultations among migrants arriving to Italy and 56.5% of consultations among the homeless populations of Paris^10^.

Scabies is more prevalent in vulnerable groups such as young children and the elderly. Eight studies have been found to directly compare scabies in adults versus children; the prevalence of scabies among children was higher in all eight studies^8^. Possible explanations for why children are disproportionally affected are overcrowding, bed sharing, under recognition, and that pruritus may be absent in infants^11^. The possible role of immune senescence in the elderly needs further research. Since the 1970’s, it has been known that impetigo is a common complication of scabies, especially in children^12^. People with scabies are reported to be 2.8 times more likely to have impetigo^13^. In Fiji and the Solomon Islands, two trials suggested the attributable risk of scabies on impetigo was between 60% and 90%^8,14^. The highest prevalence of impetigo is noted among Australian indigenous aboriginal communities, with 49% of children having impetigo^8^.

Disease burden is calculated with disability-adjusted life years (DALYs); one DALY is thought of as 1 year of healthy life lost. Scabies has been found to contribute more age-adjusted DALYs than other significant conditions such as acute lymphocytic leukaemia and arrhythmias^15^. Scabies also carries a greater burden than psoriasis and melanoma when DALYs were compared across dermatological conditions^16^. In tropical regions, the impact of this is heightened in children (age 1–4) and the geriatric population, with DALYs being lowest during adulthood^17^.

## Mite biology

Transmission occurs through skin-to-skin contact with a person who is currently infested with the scabies mite. *Sarcoptes scabiei* infects over 104 mammal species; it is thought that domestic** animals may be a source of scabies, leading to cross-species transmission^18,19^. The fertilised female mite burrows into the uninfected individual and lives for 10–14 days in the epidermis of the skin, laying up to 180 eggs in the stratum corneum^16,20^. The eggs hatch within 3 days into larvae, then become protonymphs, before becoming tritonymphs. After 4–7 days, the adult mites are present; these mites are only 0.4 mm and are not visible to the human eye^16^. There is an incubation period of 4–6 weeks where the new host may have no symptoms and be unaware that they are infested, during which they can still pass scabies onto new hosts^21^. Subsequent infestations produce symptoms within days, as the host will have developed hypersensitivity to the mite. This rapid itch and immediate scratching associated with reinfection may account for the lower scabies rates associated with adulthood.

Secondary bacterial skin infection with *Streptococcus pyogenes* can lead to septicaemia and immune-mediated disease, such as acute post-streptococcal glomerulonephritis (APSGN) and acute rheumatic fever. Scabies-related septicaemia carries a substantial mortality rate^22^. Scabies mites release complement inhibitors into the epidermis, which is thought to potentiate streptococcal and staphylococcal infections, ranging from the clinical entities of impetigo to cellulitis, abscesses, necrotising fasciitis, and finally septicaemia^8^. A hospital in Darwin, Australia, noted a 30 day mortality rate of 2.5% of all admissions to hospital with severe or crusted scabies^22^.

Evidence shows that APSGN and scabies occurrences coincide, with skin infection causing 50% of APSGN in tropical settings and an estimated 470,000 cases per year^6,23^. Treatment of scabies in communities has led to reduced levels of streptococcal skin infection and haematuria^24^. Control of scabies is crucial to prevent the development of chronic kidney disease in adulthood, a link which has been made evident in epidemiological studies and case reports^8,25^.

Recent observational studies have shown a strong association between acute rheumatic fever and the prevalence of scabies in New Zealand^26^. In Pacific island populations, there is also a strong association between permethrin prescribing, which can be seen as a marker of scabies, and acute rheumatic fever cases^26^. This would explain the high rates of rheumatic fever in certain countries where scabies and impetigo are endemic but rates of streptococcal throat are comparatively low^27^.

## Scabies diagnosis

The International Alliance for the Control of Scabies (IACS) was established in 2012, members of this group went on to produce criteria for the diagnosis of scabies in 2018, with further development in 2020 (Table 1). The IACS criteria were initially formed by a panel of 34 international experts in a Delphi consensus study^28^. It was proposed that the diagnosis can be made at one of three levels of certainty: confirmed, clinical, or suspected scabies.

**Table 1.** Summary of 2020 International Alliance for the Control of Scabies criteria for the diagnosis of scabies^21^.

**A: Confirmed Scabies**
A1: Mites, eggs, or faeces on light microscopy of skin samples A2: Mites, eggs, or faeces on individual using a high-powered imaging device A3: Mite visualised on individual using dermoscopy
**B: Clinical Scabies**
B1: Scabies burrows B2: Typical lesions affecting male genitalia B3: Typical lesions in a typical distribution and two history features
**C: Suspected Scabies**
C1: Typical lesions in a typical distribution and one history feature C2: Atypical lesions or atypical distribution and two history features
**H: History Features**
H1: Pruritus H2: Close contact with an individual who has had itch or typical lesions in a typical distribution

The table was reproduced from Engelman D *et al.* which is licensed under Creative Commons Attribution-NonCommercial-NoDerivatives 4.0 International (CC BY-NC-ND 4.0).

The established criteria can be applied to a variety of settings to facilitate diagnosis, allow comparison between research studies, and enable mapping projects and surveillance. The advantage of having different levels means that the criteria can be applied to both clinical and field settings, allowing flexibility where microscopy or thorough examination are not available. A full body examination is recommended to aid a clinical diagnosis of scabies; however, cultural beliefs and time restrictions may preclude this. Typical scabies lesions are described as more than three 2–3 mm papules or burrows that are scaly linear raised lesions^21^. A recent study revealed that examining both arms and both lower legs had a sensitivity of over 90% in comparison to examining the hands alone, which had a sensitivity of 51.2%^29^.

Limitations to the diagnostic criteria are that they do not include crusted scabies or atypical presentations; therefore, clinical judgment is still essential. Crusted scabies is a rare condition that results from an inadequate immune response to the scabies mite. These patients suffer with hyperinfestation, causing hyperkeratosis and severe inflammation. A total of 50% of patients with crusted scabies do not experience itching^30^. Table 2 shows the recommended grading scale for crusted scabies, which was developed in Darwin, Australia^31^.

**Table 2.** Clinical grading scale to guide the management of crusted scabies^31^.

**A: Distribution and extent of crusting**	
1. Wrists, web spaces, feet only (<10% total body surface area [TBSA]) 2. Above plus forearms, lower legs, buttocks, trunk, or 10–30% TBSA 3. Above plus scalp or >30% TBSA	
**B: Crusting/Shedding**	
1. Mild crusting (<5 mm depth of crust), minimal skin shedding 2. Moderate 5–10 mm crusting, moderate skin shedding 3. Severe >10 mm profuse skin shedding	
**C: Past Episodes**	
1. Never had it before 2. 1–3 prior hospitalisations for crusted scabies or depigmentation of elbows/knees 3. >4 hospitalisations for the above plus legs/back or skin thickening	
**D: Skin Condition**	
1. No cracking or pyoderma 2. Multiple pustules and/or weeping sores and/or skin cracking 3. Deep skin cracking with bleeding, widespread purulent exudates	
**Grade 1: score 4–6** **Grade 2: score 7–9** **Grade 3: score 10–12**	3 doses of ivermectin 200 μg/kg – day 0, 1, 7 5 doses – day 0, 1, 7, 8, 14 7 doses – day 0, 1, 7, 8, 14, 21, 28
All patients also treated with benzyl benzoate alternating with keratolytic cream	

This table was adapted from Davis JS *et al*.^31^ under the terms of the Creative Commons Attribution 4.0 license (CC-BY 4.0).

There are no laboratory tests readily available to confirm scabies. Instead, the “gold standard” is for mites, eggs, or faeces to be visualised using a microscope. Microscopy detection rates vary from 10 to 70%, which supports the argument for a new reliable method for diagnosing scabies^32^. Dermatoscopes have removed the necessity for invasive skin scrapings; however, dermoscopy remains operator dependent. Dermatoscopes are not affordable in all regions, cannot visualise faeces or eggs, and are harder to detect mites in darker skin types^21^. Impetigo can make the use of the dermatoscope more difficult and can spread infection^33^.

Molecular diagnostic techniques, such as PCR and loop mediated iso-thermal amplification, are currently being researched^34,35^. Nested PCR looking at the *COX1* gene has been found to be 100% sensitive compared to traditional microscopy, which is 75% sensitive^34^. Unfortunately, the detection of scabies antibodies through ELISA tests is not reliable because of similarities between scabies and house dust mite antigens causing cross-reactivity^36^. No current antibody test is available to detect previous or current exposure to scabies^7^.

## Treatment for scabies

There are effective topical and oral treatments for scabies. A 2007 Cochrane review stated that topical permethrin was the gold standard treatment for scabies. The most recent Cochrane review in 2018 showed that there was no difference in efficacy between ivermectin and permethrin; instead, treatment choices should be based on practicality, population size, licencing, and availability^37^.

Public health guidelines in Australia encourage early detection of scabies mites and 24 hours of isolation until the first treatment is carried out^38^. To prevent re-infestation, all household members and close contacts should be treated at the same time^39^. Children should return to school following the first treatment. Environmental disinfection is recommended for scabies, such as washing linen, towels, and clothing in hot water^40,41^. There is no literature available to evaluate the effectiveness of these strategies in controlling scabies; however, the UK and Australian national guidelines state that environmental strategies should not be discouraged, as they will not cause harm^42^. This is to prevent infestation, as scabies mites can live without a host for roughly 3 days^16^.

Off-host survival of the mite is sensitive to temperature and humidity conditions, with mites surviving up to a maximum of 19 days under optimal temperature and humidity conditions. For *S*. *scabiei* var. *hominis,* the longest survival occurred at 10°C and 97% relative humidity, which is a range not seen in many community settings^43^. In circumstances where hot washes are not an option, items can be kept in a sealed plastic bag for 3 days^39,41^. Environmental measures in rural village communities may not be practical due to lack of spare sheets/clothing, heavy rain fall, and no ability to dry laundry. Therefore, environmental measures were not recommended and were not used in the setting of community mass drug administration (MDA), such as the Skin Health Intervention Fiji Trial (SHIFT) in Fiji^44^. In this Fiji trial, the scabies prevalence rate went from 32.1% at baseline to 1.9%, with two treatments of ivermectin or topical permethrin cream for those with clinically diagnosed scabies, whilst single-dose ivermectin was given to other community members^44^.

### Topical treatments

Permethrin, benzyl benzoate, and sulphur-containing compounds are topical treatments available in the form of a lotion or cream. Permethrin kills larvae, nymphs, adult mites, and their eggs by affecting their nerve and muscle functions^16^. It has been noted that compliance with topical treatments is low because of the arduous task of applying the cream, repeat treatments, skin irritation, itching, malodour, and the price of the product^5,45^. Permethrin is the most effective topical treatment; however, it is relatively expensive and not available in all countries^8^.

### Systemic therapies

Ivermectin is a broad-spectrum, anti-parasitic agent which belongs to the avermectin class and causes paralysis of the parasite. Ivermectin has a short half-life of 12–56 hours and is not ovicidal; therefore, a repeat treatment is recommended once the eggs have hatched. Two studies are currently underway in the Solomon Islands and Fiji to assess if a single dose of ivermectin is as effective as two doses^46,47^.

Previous concerns surrounded the use of ivermectin during pregnancy and for small children based on data about its teratogenicity in animal studies^16,48^. However, roughly 15% of pregnant women in MDA programmes inadvertently take ivermectin, mostly in their first trimester^49^. A recent meta-analysis showed no adverse outcomes among 893 women who received ivermectin during pregnancy as part of MDA for onchocerciasis and lymphatic filariasis. There was no evidence of increased neonatal death, maternal morbidity, preterm birth, low birthweight, spontaneous abortion, or stillbirths^8,50^. Despite this, ivermectin is licensed for use in pregnancy in France only (second line) and is still not recommended in children with a body weight of <15 kg^51^. A recent multicentre observational study of 170 infants and children weighing <15 kg showed mild adverse events in seven children and no serious adverse events^52^. The AIM trial in the Solomon Islands also showed ivermectin was safe for use in children from 12.5 kg^53^. In the Skin Health Intervention Fiji Trial (SHIFT), ivermectin was associated with more adverse events than the other two treatments but all events resolved quickly^13,54^. MDA with ivermectin has an added bonus of simultaneously reducing head louse infestations; a relative reduction of 89% was seen 2 weeks after MDA in the Pacific islands. Ivermectin also reduces gastrointestinal parasites, filariasis, and onchocerciasis^55^. In June 2019, the WHO added ivermectin to the 21st WHO Essential Medicines List^51^.

### New treatments

Drugs with a longer half-life or with ovicidal properties would be revolutionary for the treatment of scabies. New anti-scabies drugs are currently being developed and trialled on porcine hosts, as pigs and humans have similar genetics, skin physiology, and immunology^32^. Two pre-clinical trials revealed a single dose of moxidectin, a highly lipophilic macrocyclic lactone, or afoxolaner, a novel acaricide, to be more effective than two doses of ivermectin^56,57^ A porcine study revealed that only 50% of 12 pigs treated with ivermectin were cured by day 14 compared to the 100% cure rate among the pigs treated with moxidectin. Moxidectin has a longer half-life and is detectable in plasma for at least 47 days compared to 7 days for ivermectin^58^. A clinical phase II trial is currently in progress to establish moxidectin as a new single-dose therapeutic (NCT03905265)^59^.

It is notable that domestic animal (primarily cat and dog) treatments for *S. scabiei* infection (termed “mange” in animals) have in recent years moved from ivermectin and moxidectin to fluralaner^60,61^. This is owing to the documented safety, efficacy, and greater duration of protection conferred by fluralaner to the host^62^. Indeed, a growing literature of fluralaner use across mammal species in the veterinary literature may suggest fluralaner could be a direction for scabies research in humans also.

## Mass drug administration

In countries in which scabies is sporadic, treatment is generally focussed on the individual and their household contacts. When scabies becomes endemic, the treatment of everyone in the community at once is found to be more effective^63^. This is called mass drug administration and can be used nationally or in confined group settings, such as schools, prisons, hospitals, and nursing homes^8^. MDA can involve topical, oral, or systemic medication. This method has successfully treated a number of diseases including filariasis, onchocerciasis, trachoma, yaws, and soil-transmitted helminths^64–67^.

Early use of MDA for scabies was demonstrated in Panama in 1991, when permethrin was used in a localised area; however, the results were not maintained^63^. In 2003, a study in the Solomon Islands gave the first indication that community-based MDA scabies treatment could not only improve quality of life but also improve haematuria^24^. SHIFT was a three-island cluster randomised controlled trial which strengthened the argument for MDA in scabies control^54^. Ivermectin was found to be the more superior treatment when compared to standard care and mass administration of permethrin^44^. The sustained reduction in scabies as well as impetigo at 24 months has confirmed its prolonged efficacy in these island-based communities^44^. A 3 year follow up after a single-round MDA of ivermectin and azithromycin in the Solomon Islands also showed the sustainability of interventions, with both scabies and impetigo rates being significantly lower than at baseline^14^. NTDs are a priority for the WHO. Owing to the effectiveness of ivermectin against a number of these NTDs, there is support for ivermectin-based MDA to be designed to treat multiple diseases at once^47^.

## Conclusion

Increased research has led WHO to classify scabies as a NTD. Scabies is endemic to underprivileged communities in tropical climates, particularly affecting children. Recent studies show that MDA is effective at reducing scabies and secondary life-threatening bacterial skin infections in countries where scabies is endemic and among institutions. The development of clinical diagnostic criteria has allowed for standardisation of the diagnosis of scabies, particularly in a clinical setting. Ivermectin and topical treatments, such as permethrin, are currently considered first line; however, this may change in coming years with the development of novel treatments.

## References

[1] World Health Organisation: Scabies and other ectoparasites. 2020. Reference Source

[2] Jackson A Heukelbach J da Silva Filho AF: Clinical features and associated morbidity of scabies in a rural community in Alagoas, Brazil. *Trop Med Int Health.* 2007; 12(4): 493–502. 10.1111/j.1365-3156.2006.01809.x 17445140

[3] Worth C Heukelbach J Fengler G: Impaired quality of life in adults and children with scabies from an impoverished community in Brazil. *Int J Dermatol.* 2012; 51(3): 275–82. 10.1111/j.1365-4632.2011.05017.x 22348561

[4] World Health Organisation: NTD-STAG Working Group on Monitoring and Evaluation of Neglected Tropical Diseases. Geneva. 2018. Reference Source

[5] La Vincente S Kearns T Connors C: Community management of endemic scabies in remote aboriginal communities of northern Australia: Low treatment uptake and high ongoing acquisition. *PLoS Negl Trop Dis.* 2009; 3(5): e444. 10.1371/journal.pntd.0000444 19478832 PMC2680947

[6] Engelman D Kiang K Chosidow O: Toward the global control of human scabies: Introducing the International Alliance for the Control of Scabies. *PLoS Negl Trop Dis.* 2013; 7(8): e2167. 10.1371/journal.pntd.0002167 23951369 PMC3738445

[7] Engelman D Cantey PT Marks M: The public health control of scabies: Priorities for research and action. *Lancet.* 2019; 394(10192): 81–92. 10.1016/S0140-6736(19)31136-5 31178154 PMC11257500

[8] Romani L Steer AC Whitfeld MJ: Prevalence of scabies and impetigo worldwide: A systematic review. *Lancet Infect Dis.* 2015; 15(8): 960–7. 10.1016/S1473-3099(15)00132-2 26088526

[9] Tjioe M Vissers WHPM: Scabies outbreaks in nursing homes for the elderly: Recognition, treatment options and control of reinfestation. *Drugs Aging.* 2008; 25(4): 299–306. 10.2165/00002512-200825040-00003 18361540

[10] Di Meco E Di Napoli A Amato LM: Infectious and dermatological diseases among arriving migrants on the Italian coasts. *Eur J Public Health.* 2018; 28(5): 910–6. 10.1093/eurpub/cky126 30010744

[11] Boralevi F Diallo A Miquel J: Clinical phenotype of scabies by age. *Pediatrics.* 2014; 133(4): e910–6. 10.1542/peds.2013-2880 24685953

[12] Bernigaud C Fischer K Chosidow O: The Management of Scabies in the 21st Century: Past, Advances and Potentials. *Acta Derm Venereol.* 2020; 100(9): adv00112. 10.2340/00015555-3468 32207535 PMC9128908

[13] Romani L Whitfeld MJ Koroivueta J: The Epidemiology of Scabies and Impetigo in Relation to Demographic and Residential Characteristics: Baseline Findings from the Skin Health Intervention Fiji Trial. *Am J Trop Med Hyg.* 2017; 97(3): 845–50. 10.4269/ajtmh.16-0753 28722612 PMC5590570

[14] Marks M Romani L Sokana O: Prevalence of Scabies and Impetigo 3 Years After Mass Drug Administration With Ivermectin and Azithromycin. *Clin Infect Dis.* 2020; 70(8): 1591–5. 10.1093/cid/ciz444 31131410 PMC7145994

[15] GBD 2015 DALYs and HALE Collaborators: Global, regional, and national disability-adjusted life-years (DALYs) for 315 diseases and injuries and healthy life expectancy (HALE), 1990–2015: A systematic analysis for the Global Burden of Disease Study 2015. *Lancet.* 2016; 388(10053): 1603–58. 10.1016/S0140-6736(16)31460-X 27733283 PMC5388857

[16] Bernigaud C Samarawickrama GR Jones MK: The Challenge of Developing a Single-Dose Treatment for Scabies. *Trends Parasitol.* 2019; 35(11): 931–43. 10.1016/j.pt.2019.08.002 31474559

[17] Karimkhani C Colombara DV Drucker AM: The global burden of scabies: A cross-sectional analysis from the Global Burden of Disease Study 2015. *Lancet Infect Dis.* 2017; 17(12): 1247–54. 10.1016/S1473-3099(17)30483-8 28941561 PMC5700804

[18] Pisano SRR Ryser-Degiorgis MP Rossi L: Sarcoptic Mange of Fox Origin in Multiple Farm Animals and Scabies in Humans, Switzerland, 2018. *Emerg Infect Dis.* 2019; 25(6): 1235–8. 10.3201/eid2506.181891 31107228 PMC6537710

[19] Fraser TA Charleston M Martin A: The emergence of sarcoptic mange in Australian wildlife: An unresolved debate. *Parasit Vectors.* 2016; 9(1): 316. 10.1186/s13071-016-1578-2 27255333 PMC4890250

[20] Thomas C Coates SJ Engelman D: Ectoparasites: Scabies. *J Am Acad Dermatol.* 2020; 82(3): 533–48. 10.1016/j.jaad.2019.05.109 31310840

[21] Engelman D Yoshizumi J Hay RJ: The 2020 International Alliance for the Control of Scabies Consensus Criteria for the Diagnosis of Scabies. *Br J Dermatol.* 2020; 183(5): 808–20. 10.1111/bjd.18943 32034956 PMC7687112

[22] Lynar S Currie BJ Baird R: Scabies and mortality. *Lancet Infect Dis.* 2017; 17(12): 1234. 10.1016/S1473-3099(17)30636-9 29173877

[23] Carapetis JR Steer AC Mulholland EK: The global burden of group A streptococcal diseases. *Lancet Infect Dis.* 2005; 5(11): 685–94. 10.1016/S1473-3099(05)70267-X 16253886

[24] Lawrence G Leafasia J Sheridan J: Control of scabies, skin sores and haematuria in children in the Solomon Islands: Another role for ivermectin. *Bull World Health Organ.* 2005; 83(1): 34–42. 15682247 PMC2623469

[25] Dowler J Wilson A: Acute post-streptococcal glomerulonephritis in Central Australia. *Aust J Rural Health.* 2020; 28(1): 74–80. 10.1111/ajr.12568 31659821

[26] Thornley S King R Marshall R: How strong is the relationship between scabies and acute rheumatic fever? An analysis of neighbourhood factors. *J Paediatr Child Health.* 2020; 56(4): 600–6. 10.1111/jpc.14697 31774599

[27] Parks T Smeesters PR Steer AC: Streptococcal skin infection and rheumatic heart disease. *Curr Opin Infect Dis.* 2012; 25(2): 145–53. 10.1097/QCO.0b013e3283511d27 22327467

[28] Engelman D Fuller LC Steer AC: Consensus criteria for the diagnosis of scabies: A Delphi study of international experts. *PLoS Negl Trop Dis.* 2018; 12(5): e0006549. 10.1371/journal.pntd.0006549 29795566 PMC5991412

[29] Marks M Engelman D Romani L: Exploration of a simplified clinical examination for scabies to support public health decision-making. *PLoS Negl Trop Dis.* 2018; 12(12): e0006996. 10.1371/journal.pntd.0006996 30589906 PMC6307692

[30] Banerji A, Canadian Paediatric Society, First Nations: Scabies. *Paediatr Child Health.* 2015; 20(7): 395–402. 10.1093/pch/20.7.395 26527041 PMC4614097

[31] Davis JS McGloughlin S Tong SYC: A novel clinical grading scale to guide the management of crusted scabies. *PLoS Negl Trop Dis.* 2013; 7(9): e2387. 10.1371/journal.pntd.0002387 24069468 PMC3772049

[32] Executive Committee of Guideline for the Diagnosis and Treatment of Scabies: Guideline for the diagnosis and treatment of scabies in Japan (third edition): Executive Committee of Guideline for the Diagnosis and Treatment of Scabies. *J Dermatol.* 2017; 44(9): 991–1014. 10.1111/1346-8138.13896 28561292

[33] Micali G Lacarrubba F Verzì AE: Scabies: Advances in Noninvasive Diagnosis. *PLoS Negl Trop Dis.* 2016; 10(6): e0004691. 10.1371/journal.pntd.0004691 27311065 PMC4911127

[34] Hahm JE Kim CW Kim SS: The efficacy of a nested polymerase chain reaction in detecting the cytochrome c oxidase subunit 1 gene of *Sarcoptes scabiei* var. *hominis* for diagnosing scabies. *Br J Dermatol.* 2018; 179(4): 889–95. 10.1111/bjd.16657 29624634

[35] Fraser TA Carver S Martin AM: A *Sarcoptes scabiei* specific isothermal amplification assay for detection of this important ectoparasite of wombats and other animals. *PeerJ.* 2018; 6: e5291. 10.7717/peerj.5291 30065882 PMC6065476

[36] Xu J Huang X Dong X: Serodiagnostic Potential of Alpha-Enolase From *Sarcoptes scabiei* and Its Possible Role in Host-Mite Interactions. *Front Microbiol.* 2018; 9: 1024. 10.3389/fmicb.2018.01024 29887838 PMC5981165

[37] Rosumeck S Nast A Dressler C: Ivermectin and permethrin for treating scabies. *Cochrane Database Syst Rev.* 2018; 4(4): CD012994. 10.1002/14651858.CD012994 29608022 PMC6494415

[38] Government VS: Scabies Control Guidelines. 2020. Reference Source

[39] Mellanby K: The development of symptoms, parasitic infection and immunity in human scabies. *Parasitology.* 1944; 35(4): 197–206. 10.1017/S0031182000021612

[40] Dermatologists BAo: Scabies Patient information leaflet. 2019; 3.

[41] CfDC, (CDC) P: Guidelines for the management of scabies. 2020. Reference Source

[42] Consortium TAHS: National Healthy Skin Guideline: for the Prevention,Treatment and Public Health Control of Impetigo, Scabies, Crusted Scabies and Tinea for Indigenous Populations and Communities in Australia. 2018. Reference Source

[43] Arlian LG Morgan MS: A review of *Sarcoptes scabiei*: Past, present and future. *Parasit Vectors.* 2017; 10(1): 297. 10.1186/s13071-017-2234-1 28633664 PMC5477759

[44] Romani L Whitfeld MJ Koroivueta J: Mass Drug Administration for Scabies - 2 Years of Follow-up. *N Engl J Med.* 2019; 381(2): 186–7. 10.1056/NEJMc1808439 31242358

[45] Currie BJ McCarthy JS: Permethrin and ivermectin for scabies. *N Engl J Med.* 2010; 362(8): 717–25. 10.1056/NEJMct0910329 20181973

[46] Lake SJ Phelan SL Engelman D: Protocol for a cluster-randomised non-inferiority trial of one versus two doses of ivermectin for the control of scabies using a mass drug administration strategy (the RISE study). *BMJ Open.* 2020; 10(8): e037305. 10.1136/bmjopen-2020-037305 32868360 PMC7462236

[47] Hardy M Samuela J Kama M: The safety of combined triple drug therapy with ivermectin, diethylcarbamazine and albendazole in the neglected tropical diseases co-endemic setting of Fiji: A cluster randomised trial. *PLoS Negl Trop Dis.* 2020; 14(3): e0008106. 10.1371/journal.pntd.0008106 32176703 PMC7098623

[48] Chaccour C Hammann F Rabinovich NR: Ivermectin to reduce malaria transmission I. Pharmacokinetic and pharmacodynamic considerations regarding efficacy and safety. *Malar J.* 2017; 16(1): 161. 10.1186/s12936-017-1801-4 28434401 PMC5402169

[49] Gyapong JO Chinbuah MA Gyapong M: Inadvertent exposure of pregnant women to ivermectin and albendazole during mass drug administration for lymphatic filariasis. *Trop Med Int Health.* 2003; 8(12): 1093–101. 10.1046/j.1360-2276.2003.01142.x 14641844

[50] Nicolas P Maia MF Bassat Q: Safety of oral ivermectin during pregnancy: A systematic review and meta-analysis. *Lancet Glob Health.* 2020; 8(1): e92–e100. 10.1016/S2214-109X(19)30453-X 31839144 PMC7613514

[51] World Health Organisation: Model List of Essential Medications, 21st List. 2019. Reference Source

[52] Levy M Martin L Bursztejn AC: Ivermectin safety in infants and children under 15 kg treated for scabies: A multicentric observational study. *Br J Dermatol.* 2020; 182(4): 1003–1006. 10.1111/bjd.18369 31344258

[53] Romani L Marks M Sokana O: Feasibility and safety of mass drug coadministration with azithromycin and ivermectin for the control of neglected tropical diseases: A single-arm intervention trial. *Lancet Glob Health.* 2018; 6(10): e1132–e1138. 10.1016/S2214-109X(18)30397-8 30223985 PMC6139784

[54] Romani L Whitfeld MJ Koroivueta J: Mass Drug Administration for Scabies Control in a Population with Endemic Disease. *N Engl J Med.* 2015; 373(24): 2305–13. 10.1056/NEJMoa1500987 26650152

[55] Coscione S Esau T Kekeubata E: Impact of ivermectin administered for scabies treatment on the prevalence of head lice in Atoifi, Solomon Islands. *PLoS Negl Trop Dis.* 2018; 12(9): e0006825. 10.1371/journal.pntd.0006825 30252856 PMC6173439

[56] Bernigaud C Fang F Fischer K: Efficacy and Pharmacokinetics Evaluation of a Single Oral Dose of Afoxolaner against *Sarcoptes scabiei* in the Porcine Scabies Model for Human Infestation. *Antimicrob Agents Chemother.* 2018; 62(9): e02334–17. 10.1128/AAC.02334-17 29914951 PMC6125498

[57] Mounsey KE Bernigaud C Chosidow O: Prospects for Moxidectin as a New Oral Treatment for Human Scabies. *PLoS Negl Trop Dis.* 2016; 10(3): e0004389. 10.1371/journal.pntd.0004389 26985995 PMC4795782

[58] Bernigaud C Fang F Fischer K: Preclinical Study of Single-Dose Moxidectin, a New Oral Treatment for Scabies: Efficacy, Safety, and Pharmacokinetics Compared to Two-Dose Ivermectin in a Porcine Model. *PLoS Negl Trop Dis.* 2016; 10(10): e0005030. 10.1371/journal.pntd.0005030 27732588 PMC5061321

[59] Ryg-Cornejo VKS: Dose-finding Study of Moxidectin for Treatment of Scabies. 2020. Reference Source

[60] Chiummo R Petersen I Plehn C: Efficacy of orally and topically administered fluralaner (Bravecto ^®^) for treatment of client-owned dogs with sarcoptic mange under field conditions. *Parasit Vectors.* 2020; 13(1): 524. 10.1186/s13071-020-04395-6 33069261 PMC7568370

[61] Kilp S Ramirez D Allan MJ: Comparative pharmacokinetics of fluralaner in dogs and cats following single topical or intravenous administration. *Parasit Vectors.* 2016; 9(1): 296. 10.1186/s13071-016-1564-8 27241240 PMC4886404

[62] Gassel M Wolf C Noack S: The novel isoxazoline ectoparasiticide fluralaner: Selective inhibition of arthropod γ-aminobutyric acid- and L-glutamate-gated chloride channels and insecticidal/acaricidal activity. *Insect Biochem Mol Biol.* 2014; 45: 111–24. 10.1016/j.ibmb.2013.11.009 24365472

[63] Taplin D Meinking TL Porcelain SL: Community control of scabies: A model based on use of permethrin cream. *Lancet.* 1991; 337(8748): 1016–8. 10.1016/0140-6736(91)92669-s 1673175

[64] WHO Guidelines Approved by the Guidelines Review Committee: Guideline: Alternative Mass Drug Administration Regimens to Eliminate Lymphatic Filariasis. Geneva: World Health Organization. Copyright (c) World Health Organization 2017. 2017. 29565523

[65] Cupp EW Sauerbrey M Richards F: Elimination of human onchocerciasis: History of progress and current feasibility using ivermectin (Mectizan(®)) monotherapy. *Acta Trop.* 2011; 120 Suppl 1: S100–8. 10.1016/j.actatropica.2010.08.009 20801094

[66] Marks M Sokana O Nachamkin E: Prevalence of Active and Latent Yaws in the Solomon Islands 18 Months after Azithromycin Mass Drug Administration for Trachoma. *PLoS Negl Trop Dis.* 2016; 10(8): e0004927. 10.1371/journal.pntd.0004927 27551787 PMC4994934

[67] Chami GF Kabatereine NB Tukahebwa EM: Profiling the best-performing community medicine distributors for mass drug administration: A comprehensive, data-driven analysis of treatment for schistosomiasis, lymphatic filariasis, and soil-transmitted helminths in Uganda. *BMC Med.* 2019; 17(1): 69. 10.1186/s12916-019-1303-z 30917824 PMC6437990

